# Streptococcus hepaticus sp. nov. isolated from the liver of domestic pigs (Sus scrofa domesticus)

**DOI:** 10.1099/ijsem.0.006776

**Published:** 2025-05-09

**Authors:** Miranda J. Kirchner, Daniel Loy, Susanna Williamson, Adrian M. Whatmore

**Affiliations:** 1 Department of Bacteriology, Animal & Plant Health Agency (Weybridge), Woodham Lane, Addlestone, Surrey, UK; 2 Animal & Plant Health Agency (Bury St. Edmunds), Rougham Hill, Bury St Edmunds, Suffolk, UK

**Keywords:** novel, pigs, species, *Streptococcus*

## Abstract

A Gram-positive coccus-shaped bacterium, which could not be identified by classical determinative bacteriology approaches, was isolated from the liver of two pigs in the United Kingdom. Initial testing based on cellular morphology and biochemical characteristics tentatively assigned the isolates to the genus *Streptococcus* but did not match any previously described species. The analysis of the 16S rRNA sequence determined that the isolates were most closely related to *Streptococcus gallinaceus* (98.6% identity). Analysis of three further housekeeping genes frequently applied in streptococcal taxonomy, *groEL*, *sodA* and *rpoB*, and a comparison against available type strain sequences confirmed that the isolates were most similar to *S. gallinaceus* in all cases (84.3%, 86.9% and 90.2% identity, respectively). The comparison of the average nucleotide identity (ANI) and *in silico* DNA–DNA hybridization values demonstrated that the novel species was distinct from other streptococcal species. Pairwise ANI values revealed that the two studied strains shared a pairwise ANI of 99.25% but were clearly distinct from previously described *Streptococcus* species (ANI ≤81.1% – best match *S. gallinaceus*). The taxonomic analysis described confirmed that the two strains represent a novel *Streptococcus* species for which the name *Streptococcus hepaticus* sp. nov. is suggested, with strain 20–1249^T^ (=NCTC 15092^T^=LMG 33498^T^) as the type strain.

The genus *Streptococcus* includes around 120 validly published species from a wide range of sources, with an increasing number of new species described in recent years (Genus: Streptococcus (bacterio.net) [1,2]. Streptococci are Gram-positive cocci, which form pairs or chains, catalase negative and facultative anaerobes. In veterinary medicine, several *Streptococcus* species are known to cause significant disease, including *Streptococcus suis*, a pathogen of pigs associated with meningitis, septicaemia and arthritis [3], *Streptococcus equi* subsp. *equi* associated with equine strangles [4] and *Streptococcus agalactiae* and *Streptococcus dysgalactiae*, which are associated with a range of diseases in farm species, including mastitis and septicaemia [5].

Two isolates of *Streptococcus,* strains 20-1249^T^ and 20–1266, were obtained from domestic pigs (*Sus scrofa domesticus)* in England in 2020. The pigs were submitted for examination to the Animal and Plant Health Agency as part of a veterinary diagnostic investigation into a disease issue in which pre-weaned piglets had been found dead. Both isolates originated from the same farm premises but were obtained from two different individual pigs 3 months apart. Alpha-haemolytic streptococci were recovered in moderate pure growth from liver swabs taken from dead pigs at post-mortem examination. The swabs were cultured on 5% sheep-blood agar (SBA) at 37 °C. Swabs were collected by insertion into the liver parenchyma after the liver surface had been seared. There was no evidence of histopathological changes in the liver consistent with septicaemia in the pig from which 20-1249^T^ was isolated. No histopathology was undertaken on the liver of the second pig. Both pigs were found to have primary intestinal disease, and coccidiosis due to *Cystoisospora suis* was diagnosed in the second submission. Isolates had identical morphologies on SBA, with evidence of alpha-haemolysis. Standard microbiological methods, including growth on MacConkey agar and aesculin blood agar, Gram-staining, catalase testing and Analytical profile index (API) Rapid ID 32 Strep were unable to determine the species. Molecular and biochemical analyses were carried out to identify and characterize these isolates which is described here.

The virtually complete 16S rRNA gene sequences were determined for both isolates using previously described methods [6]. The 16S rRNA gene sequence of 20-1249^T^ has been deposited in GenBank with accession no. OR166132. Across a fragment of 1,477 bp, both isolates have identical sequence. The top BLASTn (https://www.ncbi.nlm.nih.gov/geo/query/blast.html) matches to extant streptococcal species are with *Streptococcus gallinaceus* (NR_025453.1 : 98.58%), followed by *Streptococcus koreensis* (CP032620.1 : 97.1%), *Streptococcus respiraculi* (CP022680.1 : 96.83%), *S. suis* (LS483418.1 : 96.76%) *and Streptococcus marmotae* (NR_152678.1 : 96.62%) [7,11]. Of note, the sequence of 20-1249^T^ and of 20–1266 is 100% identical to a 16S rRNA gene database entry (1,479 bp: AB826496) from an undescribed *Streptococcus* isolated from pig liver in Japan (Strain Str-88). The phylogenetic analysis of the 16S rRNA gene sequence was performed with selected streptococcal species. BLASTn [12] analysis (with type strains) identified streptococcal species with >95 % sequence identity, which were aligned using clustal in mega X [13,14], and mega X was also used to generate a phylogenetic tree with a neighbour-joining approach. This demonstrated that 20-1249^T^ and 20–1266, and the identical sequence from Str-88 form a well-supported group most closely related to *S. gallinaceus* (Fig. 1).

**Fig. 1.**
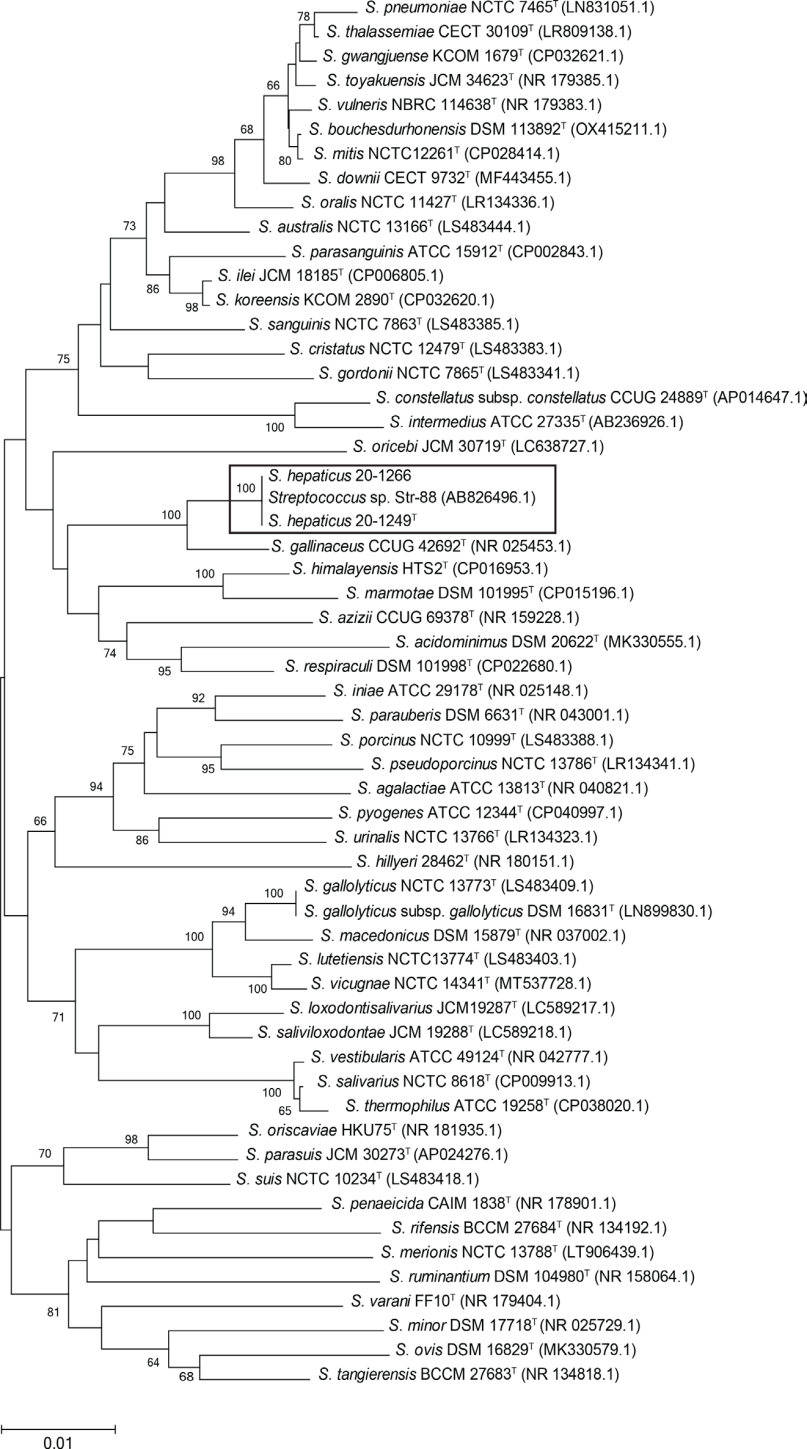
Unrooted tree demonstrating the phylogenetic relationship generated from 16S rRNA gene sequences of *Streptococcus* species most closely related to 20-1249^T^. Analysis was carried out using the MEGA neighbour-joining tree and evolutionary distances calculated with Jukes–Cantor and 100 bootstraps. Sequences were trimmed to a consensus of 1,477 bp and included type strains, the sequence of isolate Str88 (AB826496.1) and both *Streptococcus hepaticus* sp. nov. isolates. GenBank accession numbers are shown in brackets. Values at nodes correspond to proportions of 100 resamplings that support the topology shown, with only values >60% indicated. Bar, 0.01 substitutions per nucleotide position. *S. hepaticus* sp. nov and the Str-88 isolate are highlighted with a box.

The relatedness of isolates was determined by whole-genome sequence (WGS) analysis of both isolates. The data were then compared to published genomes from other streptococcal species. DNA was extracted using the KingFisher automated DNA isolation system (ThermoFisher) and sequenced using an Illumina NextSeq with coverage ranging from 257 to 329 x. Raw reads were assembled using Unicycler into contigs between 57 and 73 [15]. The G and C content of 20-1249 was determined to be 40.7 mol%, which is within the range (33.79% and 43.40%) previously reported for streptococcal species [16]. The assembled genome sequence of 20-1249^T^ is available in National Center for Biotechnology Information (NCBI, https://www.ncbi.nlm.nih.gov/) within the project PRJNA985899.

The sequences of housekeeping genes, *rpoB*, *sodA* and *groEL*, frequently used in taxonomic placement of streptococci, were extracted from the WGS data to determine the relatedness to other streptococcal species [17,18]. The individual sequences of these housekeeping genes were deposited with accession numbers: (*rpoB*: OR160292), (*sodA*: OR160293) and (*groEL*: OR160294). The sequences for each gene were identical between isolates for *rpoB* and *sodA,* and there was a single nucleotide substitution within the *groEL* contig. The *rpoB* (contig size=638 bp), *sodA* (contig size=380 bp) and *groEL* (contig size=752 bp) from 20-1249^T^ were compared with the NCBI sequence database using BLASTn, and streptococcal sequences from type strains were included in analyses. Sequences were excluded when the sequence identity was <80% for *rpoB* and *groEL*, and <75% for *sodA*. Sequences were aligned using clustal and phylogenetic comparison performed in mega X. For all genes, phylogenetic analysis determined the closest related extant streptococcal species was *S. gallinaceus*, which had 84.3% (*groEL*), 86.9% (*sodA*) and 90.2% (*rpoB*) identity compared to 20-1249^T^ (Supplementary information Figs S2–S4, respectively, are available with the online version of this article). The sequences of these genes also matched closely with the unpublished porcine *Streptococcus* sp. Str-88, with *sodA* having one SNP across 380 bp, and *groEL* and *rpoB*, having 0 SNPs across the 752 bp and 638 bp analysed, respectively (*rpoB*: AB827270, *sodA*: AB827271 and *groEL*: AB851477). Taken together, the phylogenetic analysis of all three housekeeping genes and the 16S rRNA gene shows that strains 20-1249^T^ and 20–1260 form a distinct taxon within the genus *Streptococcu*s, with the closest related extant species being *S. gallinaceus,* a species most commonly associated with poultry [7,19] and occasional zoonotic infection of occupationally exposed individuals [20].

*In silico* DNA–DNA hybridization (isDDH) was performed using the Genome-to-Genome Distance Calculator (formula 2) (https://ggdc.dsmz.de/). The isDDH values were between 22% and 29% when the genome sequence of 20-1249^T^ was compared to closely related streptococcal species (Table 1). These values are below the expected cut-off values for defining a species (≥70%) [21]. The average nucleotide identity (ANI) was also determined using J Species (https://jspecies.ribohost.com/jspeciesws/) [22]. ANI was calculated for closely related streptococcal species which were available in Table 1. The most closely related genome using ANI is from *S. gallinaceus*, with an ANIb value of 81%. However, this is considerably below the threshold of ≥95–96%, which is used to define a species [23] confirming the identity of these strains as a novel taxon within the genus *Streptococcus*.

**Table 1.** ANI and isDDH values for isolate 20-1249^T^ compared to other published streptococcal species and the other novel isolate (20-1266)

**Species**	Accession no.	ANIb (%)	isDDH (%)
20-1266		99.25	96
Streptococcus. gallinaceus ^*^ (DSM 15349^T^)	IMG ref 252529	81.12	24.4
*Streptococcus suis* (NCTC 10234^T^)	GCA_000294495	73.56	22.8
*Streptococcus porci* (DSM 23759^T^)	GCA_000423765	73.4	28.8
*Streptococcus minor* (DSM 17118^T^)	GCA_000377005	72.90	22.4
*Streptococcus equinus* (ATCC 9812^T^)	GCA_000187265	71.70	23
*Streptococcus parasanguinis* (ATCC 15912^T^)	GCA_000164675	71.31	22.8
*Streptococcus australis* (ATCC 700641^T^)	GCA_000186465	71.23	22.3
*Streptococcus sanguinis* (NCTC 7863^T^)	GCA_000194945	70.97	23.5
*Streptococcus criceti* (NCTC 12277^T^)	GCA_000187975	70.33	23.8

*S. gallinaceus genome sequence deposited in Intergrated microbial genomes/microbiomes (IMG; https://img.jgi.doe.gov/).

Both strains were fully characterized using API Rapid ID32 Strep and API 50CH according to the manufacturer’s instructions (bioMerieux). The Streptococcal Grouping Kit (Thermo Fisher) was utilized to determine the Lancefield serogroup for both strains. However, a Lancefield group could not be determined for these isolates. Several biochemical tests were identified, which could be used to distinguish 20-1249^T^ from other closely related streptococcal species (Table 2).

**Table 2.** Biochemical characteristics used to distinguish *S. hepaticus* sp. nov from closely related species [24]

Test	*S. hepaticus sp. nov*	*S. gallinaceus*	*S. suis*	*S. minor*	*S. porci* [25]
d-Sorbitol	+	−	−	d	−
d-Raffinose	+	+	d	d	+
d-Ribose	+	+	−	−	−
d-Melibiose	−	+	d	−	+
β-Mannosidase	+	−	d	nd	−
β-Glucuronidase	−	−	+	−	−
Lancefield grouping	NG	D, NG	R, S, T, NG	NG	B

d, variable result; nd, Not done; NG, no Lancefield group.

Molecular and biochemical analyses of these isolates indicate that they represent a novel bacterial species within the genus *Streptococcus*, for which we propose the name *S. hepaticus* sp. nov. The strains appear to reflect incidental isolation in pigs with intestinal disease, and any role in animal disease remains undetermined. While the original strains were isolated in the UK, the identification of an identical sequence deposited in GenBank from the same anatomical site and host species in Japan suggests a wider global distribution of *S. hepaticus* sp. nov.

## Description of *Streptococcus hepaticus* sp. nov.

*Streptococcus hepaticus* (he.pa’ti.cus. N.L. masc. adj. *hepaticus* (from Gr. masc. adj. *hepatikos*) of the liver, from which the bacterium was first isolated).

The cells of *S. hepaticus* are Gram-positive cocci-shaped bacteria. Colonies are small (∼1.0 mm in diameter) circular colonies, which are non-pigmented after growth on SBA for 24 h at 37 °C and are alpha-haemolytic. Growth is achieved under aerobic and anaerobic conditions. *S. hepaticus* can grow on Tryptone-soy agar containing 5% sheep blood, and Columbia agar supplemented with 10 mg l^−1^ Colistin and 15 mg l^−1^ nalidixic acid (Colistin-Nalidixic acid CNA supplement) and 5% horse blood. *S. hepaticus* was unable to grow on MacConkey agar and Man, Rogosa, Sharpe agar. Lancefield grouping was unable to determine any serogroup. Isolates were indole, catalase and oxidase negative. Testing with API kits determined that acid was produced with cellobiose, fructose, galactose, glucose, inulin, lactose (bovine origin), maltose, mannitol, mannose, melibiose, methyl-β-d-glucoside, N-acetyl-glucosamine, ribose, raffinose, saccharose, sorbitol, salicin and trehalose (48 h). A variable reaction was obtained for acid production from aesculin, glycogen and pullulan between the two isolates, although 20-1249^T^ was negative for acid production in these tests. Acid was not produced from arabinose, arabitol (d- and l-), amygdalin, arbutin, cyclodextrin, dulcitol, erythritol, fucose (d- and l-), gentibiose, glycerol, inositol, lyxose, melezitose, rhamnose, sorbose, tagatose, turanose, xylitol and xylose (d- and l-). The activity was observed for β-glucosidase, α-galactosidase, β-galactosidase, β-mannosidase, arginine dihydrolase, alanyl-phenylalanyl-proline arylamidase and glycyl-tryptophan arylamidase, but the activity of β-glucuronidase, pyroglutamic acid arylamidase and N-acetyl-β-glucosaminidase was not observed. The novel species was unable to produce acetoin (Voges–Proskauer negative), unable to hydrolyse hippurate and urease negative. The G and C content of the DNA is 40.7 mol%. The sequence of the 16S rRNA gene and the genome sequence of the strain 20-1249^T^ have the accession numbers OR166132 and JBINIY000000000, respectively. The type strain is NCTC 15092^T^=LMG 33498^T^=20–1249^T^. *S. hepaticus* was isolated from swabs taken from the livers of pre-weaned domestic pigs in the United Kingdom.

## Supplementary material

10.1099/ijsem.0.006776 Uncited Supplementary Material 1.

## Abbreviations

ANI: average nucleotide identity
DDH: DNA–DNA hybridization
SBA: sheep-blood agar
WGS: whole-genome sequence
