# Supplementary material for: Streptococcus hepaticus sp. nov. isolated from the liver of domestic pigs (Sus scrofa domesticus)
Source: Int J Syst Evol Microbiol. 2025 May 9;75(5):006776. doi: 10.1099/ijsem.0.006776 (PMC12064851; doi:10.1099/ijsem.0.006776)
Supplement: Uncited Supplementary Material 1. [file ijsem-75-06776-s001.pdf]

Figure 2.

Phylogenetic analysis of *groEL* carried out with MEGA x using the neighbour-joining approach with evolutionary distances computed using the Jukes-Cantor with a contig of 752bp. Analysis of the *S. hepaticus* sp. nov. *groEL* contig using BLASTn identified closely related sequences which were used to generate this tree. Sequences from type strains that had >80% sequence identity were included along with Str-88 (AB851477.1) and both *S. hepaticus* sp. nov. isolates (20-1249^T and 20-1266). Value at nodes correspond to proportions of 100 re-samplings that support the topology shown with only values >60% indicated. Bar, 0.02 substitutions per nucleotide position. Sequence accession numbers are shown in brackets.

Figure 2.

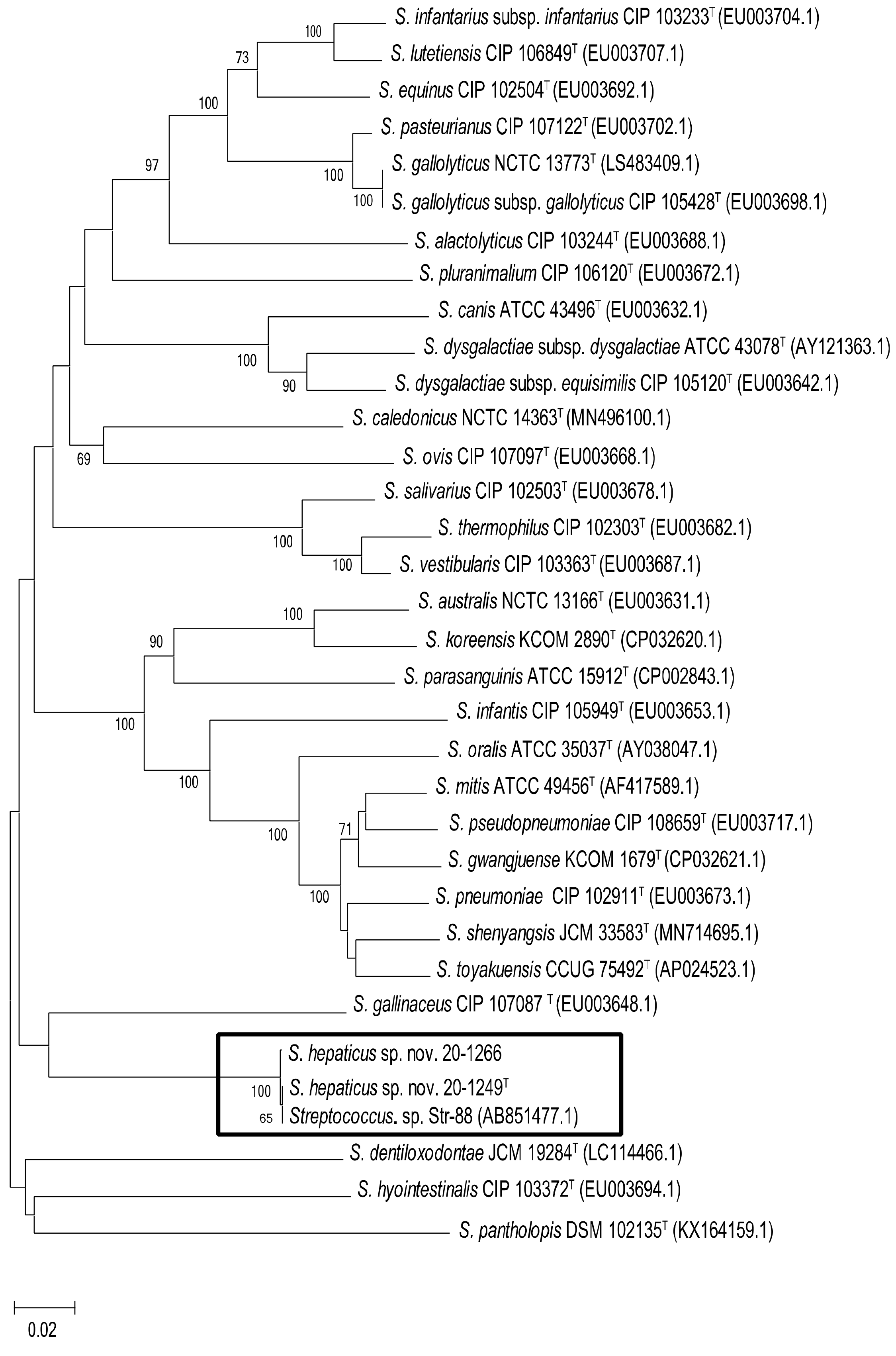

S. infantarius subsp. infantarius CIP 103233T (EU003704.1)
S. lutetiensis CIP 106849T (EU003707.1)
S. equinus CIP 102504T (EU003692.1)
S. pasteurianus CIP 107122T (EU003702.1)
S. gallolyticus NCTC 13773T (LS483409.1)
S. gallolyticus subsp. gallolyticus CIP 105428T (EU003698.1)
S. alactolyticus CIP 103244T (EU003688.1)
S. pluranimalium CIP 106120T (EU003672.1)
S. canis ATCC 43496T (EU003632.1)
S. dysgalactiae subsp. dysgalactiae ATCC 43078T (AY121363.1)
S. dysgalactiae subsp. equisimilis CIP 105120T (EU003642.1)
S. caledonicus NCTC 14363T (MN496100.1)
S. ovis CIP 107097T (EU003668.1)
S. salivarius CIP 102503T (EU003678.1)
S. thermophilus CIP 102303T (EU003682.1)
S. vestibularis CIP 103363T (EU003687.1)
S. australis NCTC 13166T (EU003631.1)
S. koreensis KCOM 2890T (CP032620.1)
S. parasanguinis ATCC 15912T (CP002843.1)
S. infantis CIP 105949T (EU003653.1)
S. oralis ATCC 35037T (AY038047.1)
S. mitis ATCC 49456T (AF417589.1)
S. pseudopneumoniae CIP 108659T (EU003717.1)
S. gwangjuense KCOM 1679T (CP032621.1)
S. pneumoniae CIP 102911T (EU003673.1)
S. shenyangsis JCM 33583T (MN714695.1)
S. toyakuensis CCUG 75492T (AP024523.1)
S. gallinaceus CIP 107087 T (EU003648.1)
S. hepaticus sp. nov. 20-1266
S. hepaticus sp. nov. 20-1249T
Streptococcus. sp. Str-88 (AB851477.1)
S. dentiloxodontae JCM 19284T (LC114466.1)
S. hyointestinalis CIP 103372T (EU003694.1)
S. pantholopis DSM 102135T (KX164159.1)
100
73
100
97
100
100
100
90
69
100
100
100
90
100
100
100
71
100
100
65
0.02

Figure 3.

Phylogenetic analysis of *sodA* carried out with MEGA X using the neighbour-joining approach with evolutionary distances computed using the Jukes-Cantor with a contig of 380bp. Analysis of the *S. hepaticus* sp. nov. *sodA* contig using BLASTn identified closely related sequences which were used to generate this tree. Sequences from type strains that had >75% sequence identity were included. The tree includes the *sodA* sequence of isolate Str88 (AB827271.1) and both *S. hepaticus* sp. nov. strains (20-1249^{T} and 20-1266). Value at nodes correspond to proportions of 100 re-samplings that support the topology shown with only values >60% indicated. Bar, 0.05 substitutions per nucleotide position.

Figure 3.

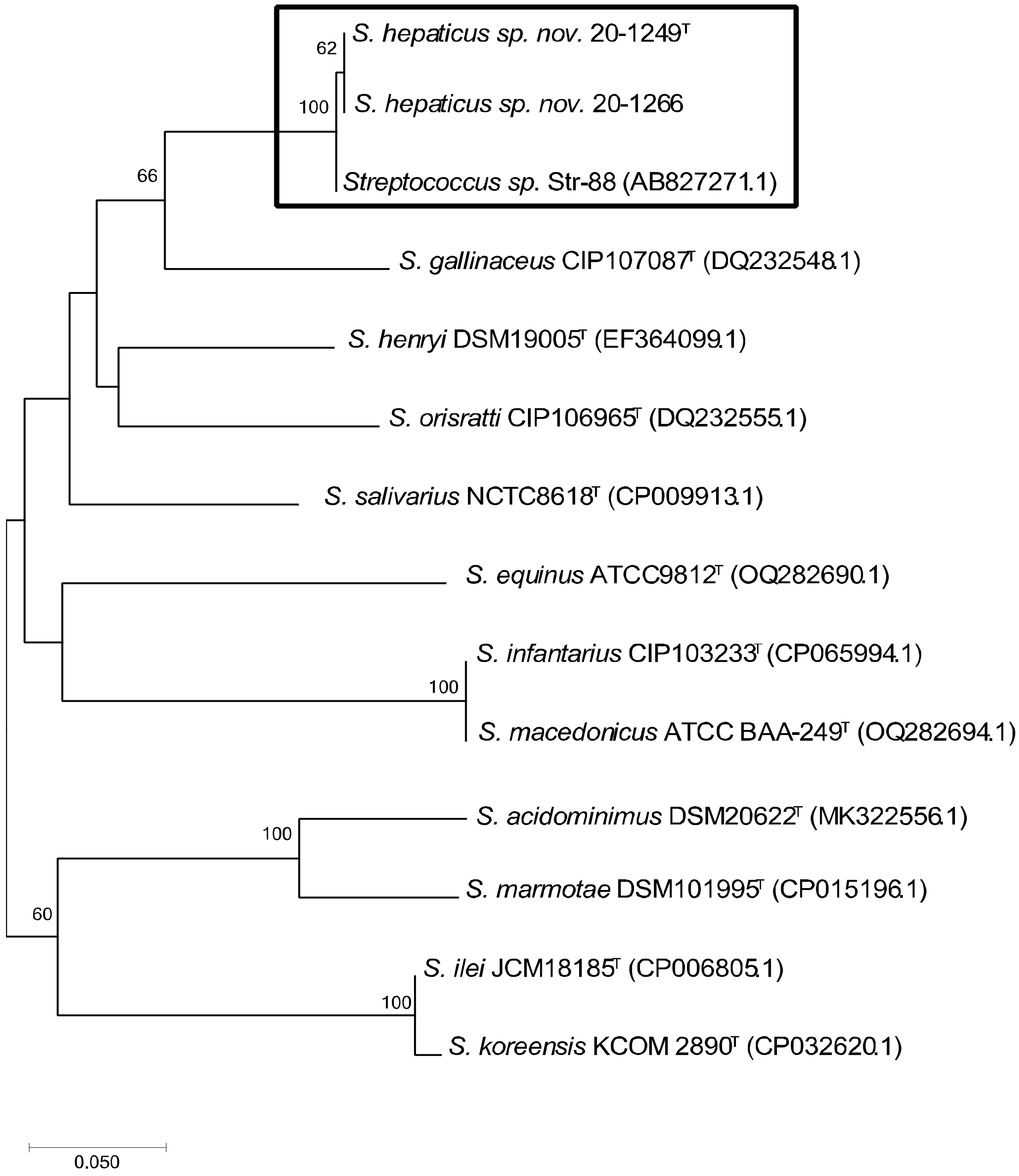

62
S. hepaticus sp. nov. 20-1249T
100
S. hepaticus sp. nov. 20-1266
66
Streptococcus sp. Str-88 (AB827271.1)
S. gallinaceus CIP107087T (DQ232548.1)
S. henryi DSM19005T (EF364099.1)
S. orisratti CIP106965T (DQ232555.1)
S. salivarius NCTC8618T (CP009913.1)
S. equinus ATCC9812T (OQ282690.1)
S. infantarius CIP103233T (CP065994.1)
100
S. macedonicus ATCC BAA-249T (OQ282694.1)
S. acidominimus DSM20622T (MK322556.1)
100
S. marmotae DSM101995T (CP015196.1)
60
S. ilei JCM18185T (CP006805.1)
100
S. koreensis KCOM 2890T (CP032620.1)
0.050

Figure 4.

Phylogenetic analysis of *rpoB* carried out with MEGA x using the neighbour-joining approach with evolutionary distances computed using the Jukes-Cantor with a contig of 638bp. Analysis of the *S. hepaticus* sp. nov. *rpoB* contig using BLASTn identified closely related sequences which were used to generate this tree. Sequences from type strains that have >80% sequence identity were included along with the contig from Str-88 (AB827270.1) and both *S. hepaticus sp. nov.* strains (20-1249^{T} and 20-1266). Value at nodes correspond to proportions of 100 re-samplings that support the topology shown with only values >60% indicated. Bar, 0.02 substitutions per nucleotide position.

Figure 4.

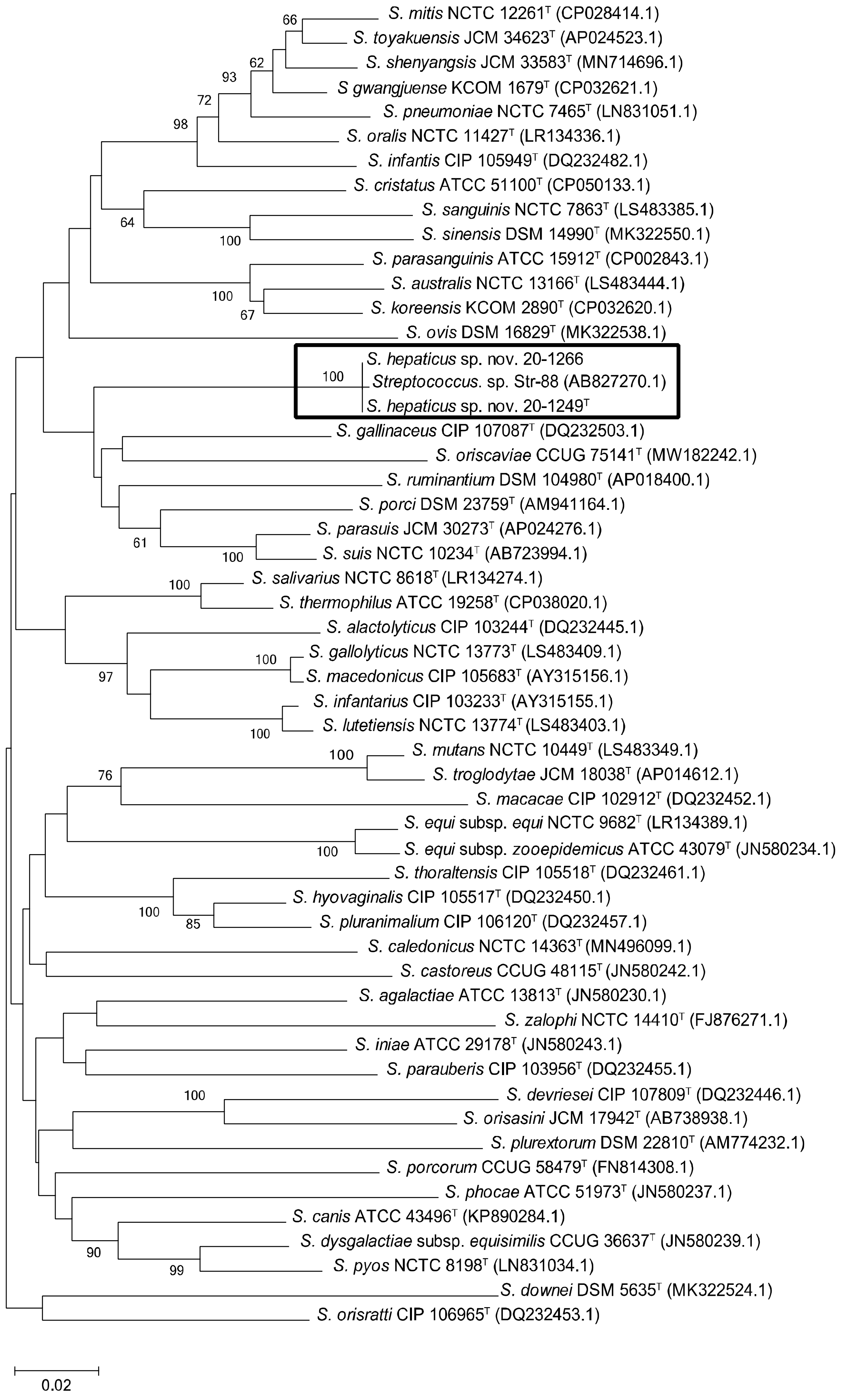
S. mitis NCTC 12261T (CP028414.1)
S. toyakuensis JCM 34623T (AP024523.1)
S. shenyangsis JCM 33583T (MN714696.1)
S gwangjuense KCOM 1679T (CP032621.1)
S. pneumoniae NCTC 7465T (LN831051.1)
S. oralis NCTC 11427T (LR134336.1)
S. infantis CIP 105949T (DQ232482.1)
S. cristatus ATCC 51100T (CP050133.1)
S. sanguinis NCTC 7863T (LS483385.1)
S. sinensis DSM 14990T (MK322550.1)
S. parasanguinis ATCC 15912T (CP002843.1)
S. australis NCTC 13166T (LS483444.1)
S. koreensis KCOM 2890T (CP032620.1)
S. ovis DSM 16829T (MK322538.1)
S. hepaticus sp. nov. 20-1266
Streptococcus. sp. Str-88 (AB827270.1)
S. hepaticus sp. nov. 20-1249T
S. gallinaceus CIP 107087T (DQ232503.1)
S. oriscaviae CCUG 75141T (MW182242.1)
S. ruminantium DSM 104980T (AP018400.1)
S. porci DSM 23759T (AM941164.1)
S. parasuis JCM 30273T (AP024276.1)
S. suis NCTC 10234T (AB723994.1)
S. salivarius NCTC 8618T (LR134274.1)
S. thermophilus ATCC 19258T (CP038020.1)
S. alactolyticus CIP 103244T (DQ232445.1)
S. gallolyticus NCTC 13773T (LS483409.1)
S. macedonicus CIP 105683T (AY315156.1)
S. infantarius CIP 103233T (AY315155.1)
S. lutetiensis NCTC 13774T (LS483403.1)
S. mutans NCTC 10449T (LS483349.1)
S. troglodytae JCM 18038T (AP014612.1)
S. macacae CIP 102912T (DQ232452.1)
S. equi subsp. equi NCTC 9682T (LR134389.1)
S. equi subsp. zooepidemicus ATCC 43079T (JN580234.1)
S. thoraltensis CIP 105518T (DQ232461.1)
S. hyovaginalis CIP 105517T (DQ232450.1)
S. pluranimalium CIP 106120T (DQ232457.1)
S. caledonicus NCTC 14363T (MN496099.1)
S. castoreus CCUG 48115T (JN580242.1)
S. agalactiae ATCC 13813T (JN580230.1)
S. zalophi NCTC 14410T (FJ876271.1)
S. iniae ATCC 29178T (JN580243.1)
S. parauberis CIP 103956T (DQ232455.1)
S. devriesei CIP 107809T (DQ232446.1)
S. orisasini JCM 17942T (AB738938.1)
S. plurextorum DSM 22810T (AM774232.1)
S. porcorum CCUG 58479T (FN814308.1)
S. phocae ATCC 51973T (JN580237.1)
S. canis ATCC 43496T (KP890284.1)
S. dysgalactiae subsp. equisimilis CCUG 36637T (JN580239.1)
S. pyos NCTC 8198T (LN831034.1)
S. downei DSM 5635T (MK322524.1)
S. orisratti CIP 106965T (DQ232453.1)
66
62
93
72
98
64
100
100
67
100
61
100
100
100
97
100
100
76
100
100
85
100
90
99
0.02
